# Calcium binding by γ-carboxyglutamic acid: it takes two to tether

**DOI:** 10.1016/j.rpth.2025.102964

**Published:** 2025-06-27

**Authors:** Hans Ippel, Sem J. Peijnenborgh, Tilman M. Hackeng, Stijn M. Agten

**Affiliations:** Department of Biochemistry, Cardiovascular Research Institute Maastricht (CARIM), University Maastricht, Maastricht, the Netherlands

**Keywords:** blood coagulation factors, calcium-binding proteins, carboxylic acids, glutamic acid, vitamin K

## Abstract

**Background:**

The small family of vitamin K-dependent proteins are characterized by posttranslational modification of specific glutamic acid residues to yield γ-carboxyglutamic acid (Gla). Gla residues give these proteins calcium ion-binding properties, which are essential for a number of coagulation factors and mineralization processes. Biophysical characteristics of Gla are, however, incomplete, hindering molecular dynamics simulations and protein structure predictions.

**Objectives:**

This study aimed to elucidate the general biophysical characteristics (p*K*_a_ and *K*_D_) of calcium binding to γ-carboxyglutamic acid in a protein environment and determine how positioning of γ-carboxyglutamic acid influences cooperative calcium binding and protein structure.

**Methods:**

Residue-based p*K*_a_ of Gla carboxyl groups in model peptides was individually determined by measuring ^1^H and ^13^C nuclear magnetic resonance chemical shift changes as a function of pH. In addition, residue-based *K*_D_ values of Ca^2+^ binding were determined using Ca^2+^ nuclear magnetic resonance titrations. Secondary structure of peptides and proteins was assessed using circular dichroism and nuclear magnetic resonance.

**Results:**

Carboxylic acid groups present on Gla residues have 2 different p*K*_a_ values of 2.62 ± 0.07 and 5.02 ± 0.05. In presence of calcium ions, p*K*_a_ values drop to 2.54 ± 0.02 and 4.55 ± 0.04. Affinity of a single Gla residue for calcium is low (∼15 mM); 2 Gla residues show cooperativity, resulting in a 25-fold increased affinity for calcium ions (0.6 mM). Finally, cooperative calcium ion binding led to increased α-helical content in model proteins.

**Conclusion:**

Vitamin K-dependent proteins present Gla residues in a different manner but benefit from cooperative calcium ion binding. Experimentally determined p*K*_a_ and *K*_D_ values can be used for interpretation of binding interactions or for molecular dynamics simulations of Gla domains with unknown structure.

## Essentials

• γ-Carboxyglutamic acid (Gla) is an essential amino acid for coagulation and mineralization.
• Biophysical characteristics of Gla are not completely known.
• Gla has 2 different p*K*a values that decrease in presence of calcium.
• Two Gla residues are required for optimal calcium binding.

## 1 Introduction

Posttranslational carboxylation of glutamic acid (Glu) yields γ-carboxyglutamic acid (Gla), an amino acid with a dicarboxylic acid and Ca^2+^-binding capabilities. Glu is converted to Gla via vitamin K-dependent carboxylation [[1], [2], [3]].

Proteins containing Gla amino acids are referred to as vitamin K-dependent (VKD) proteins. The most well-known are coagulation factors such as prothrombin and factor (F)VII, FIX, and FX and anticoagulants such as protein S, protein C, and protein Z. However, VKD proteins also include proteins associated with mineralization such as matrix Gla protein, osteocalcin, and Gla-rich protein (GRP) [4]. Gla residues are vital for the function of VKD proteins through their Ca^2+^-binding capabilities. However, mechanisms by which individual Gla residues bind calcium ions or how secondary structure influences Ca^2+^ binding are not well understood. In addition, acid dissociation constants (p*K*_a_) for Gla in a protein environment have not been experimentally determined. These biophysical characteristics are of great importance to molecular modeling approaches as they may be used to predict protein structure and function.

**Figure 1:**
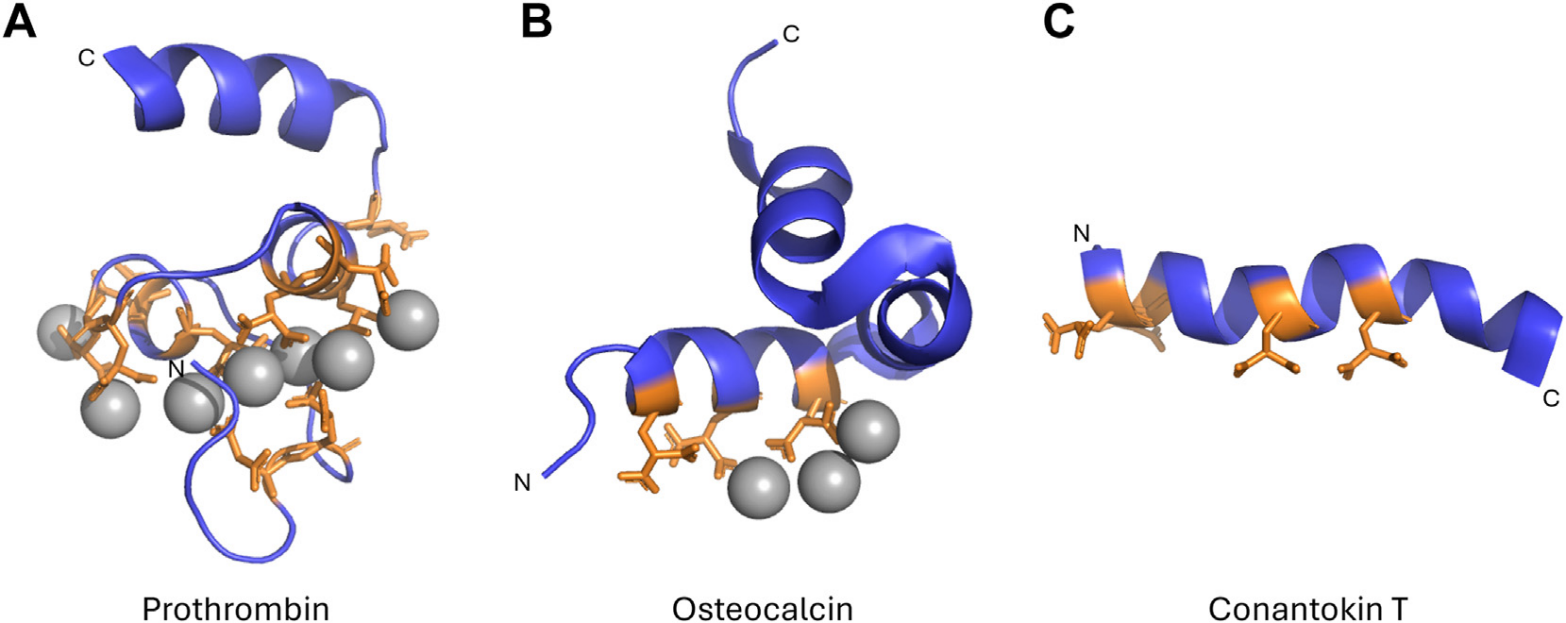
Molecular structures of (A) γ-Carboxyglutamic acid (Gla)-domain of prothrombin (Protein Data Bank (PDB): 2PF2), (B) osteocalcin (PDB: 1Q8H) and (C) conantokin T (1ONT). Gla residues are shown as sticks in orange and calcium ions as gray balls, and N-terminus and C-terminus are denoted [[11], [12], [13]].

**Figure 2:**
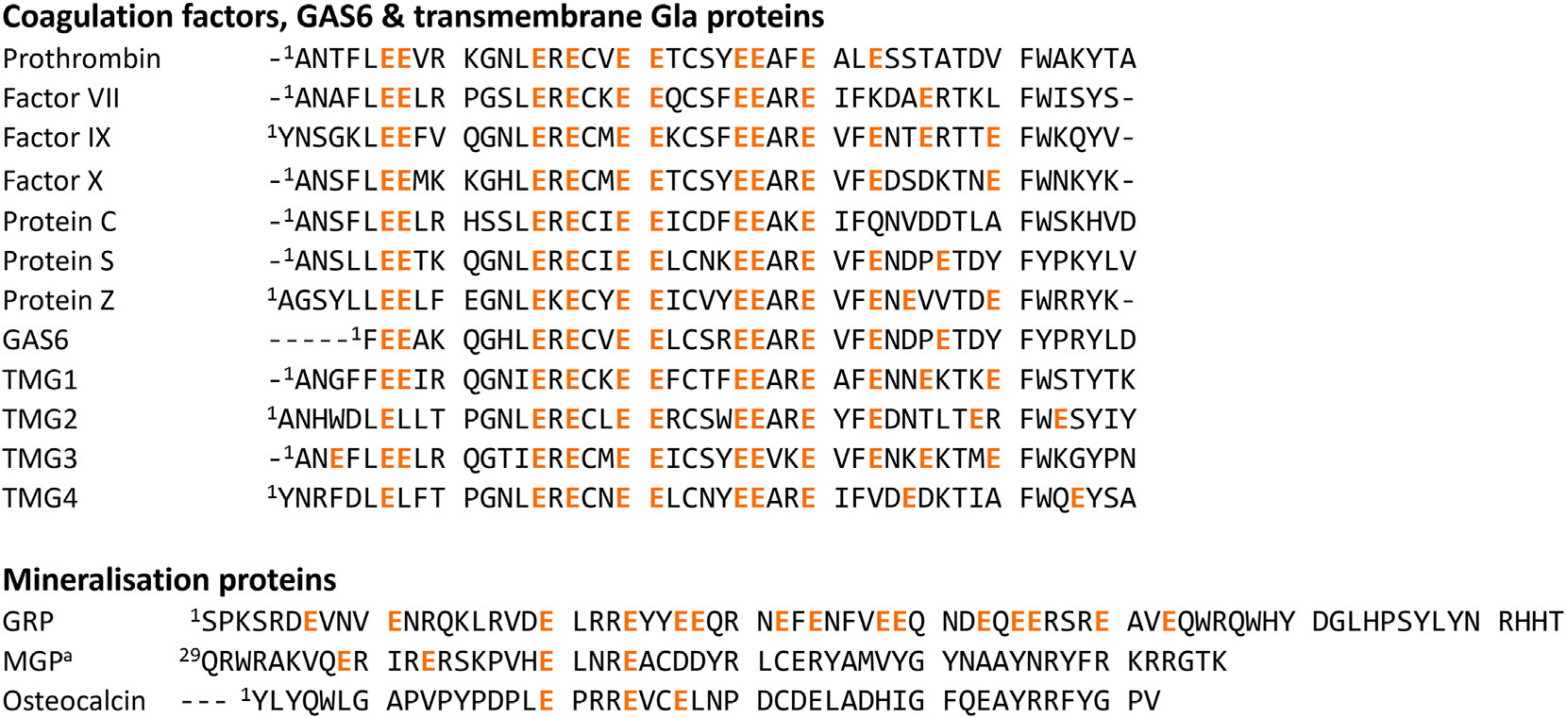
Multiple sequence alignment (MSA) of γ-carboxyglutamic acid (Gla) domains of coagulation factors, Gas6, transmembrane Gla proteins, and mineralization proteins (all of human origin). Results shown are the result of 2 separate MSAs using Clustal Omega. Highlighted glutamic acid residues are posttranslationally modified to Gla. ^a^For clarity, the first 28 residues from matrix Gla protein (MGP) were omitted, position 2 in MGP contains an additional Gla residue.

VKD proteins involved in blood coagulation have highly homologous N-terminal Gla domains containing 9–13 Gla residues optimally spaced *(i*, *i* + 1 and *i*, *I* + 2) to ensure Ca^2+^ binding in a compact globular fold [[5], [6], [7]] (Figures 1A and 2). Upon Ca^2+^ binding, a structural change is induced, leading to the exposure of phospholipid membrane-binding (exo)sites [8,9]. The location of Ca^2+^ binding is highly conserved among the Gla domains of coagulation factors [10]. In contrast to coagulation factors, bone mineralization VKD proteins such as osteocalcin and matrix Gla protein have very different Gla domain sequences. In this case, Gla residues are spaced in (*i*, *i* + *3* or *i*, *i* + *4*) motifs that present Gla residues on 1 side of an α-helix (Figures 1B and 2) [[11], [12], [13]]. Specific Gla spacing may make binding to a calcium phosphate crystal lattice more favorable for the bone mineralization group of VKD proteins whereas the spacing in the coagulation group of proteins may be better suited for binding calcium ions.

Crystal or nuclear magnetic resonance (NMR) structures available for osteocalcin, FIX, FX and prothrombin show multiple Gla residues are involved in binding a single Ca^2+^ ion with an apparent ratio of 2 Gla residues per calcium ion [6,7,[10], [11], [12]]. This may suggest cooperativity in Ca^2+^ binding. Previous studies have shown Gla residues in protein C have different affinities for calcium, with high and low affinity–binding sites [14,15]. Moreover, binding affinities of some coagulation factor Gla domains for phospholipid membranes are known [16,17].

Previous studies have shown that side-chain carboxylic acids in Gla residues may have 2 different p*K*_a_ values, similar to other dicarboxylic or tetracarboxylic acids such as methylmalonic acid or EDTA [1]. In addition, the protonation state of Gla may be influenced by the surrounding protein structure as well as calcium binding. An early study has looked at p*K*_a_ values of isolated D,L-Gla residues [2] but detailed studies of Gla in a protein environment, including effects of neighboring Gla residues or calcium ions, are lacking.

We hypothesize that Ca^2+^ binding by Gla residues is cooperative and influenced by protein secondary structure. We therefore synthesized multiple model peptides incorporating Gla in random coil or defined secondary structure. Residue-based p*K*_a_ of each Gla carboxyl group was individually determined by measuring ^1^H and ^13^C chemical shift changes as a function of pH. In addition, dissociation constants (*K*_D_) of Ca^2+^ binding were determined using a Ca^2+^ titration using NMR ^13^C spectroscopy. Secondary structure was determined using a combination of circular dichroism (CD) and NMR spectroscopy.

## 2 Materials and Methods

### 2.1 Fmoc synthesis of peptides

Rink amide resin (0.05 mmol) was swelled in dimethylformamide (DMF) (5 mL, 1 hour) and subsequently in dichloromethane (DCM) (5 mL, 1 hour). Initial Fmoc group was deprotected using 20% piperidine in DMF (2 mL) in a mechanical shaker (2 × 5 mins). Next, resin was washed with DMF (5 × 2 mL) and DCM (5 × 2 mL). Amino acids were subsequently iteratively coupled using the following steps: in short, the resin was washed with DMF (5 × 2 mL) and DCM (5 × 2 mL), and the Fmoc group of resin-bound amino acid was removed with 20% piperidine in DMF (2 × 2 mL × 5 minutess) with mechanical shaking, followed by resin washing with DMF (5 × 2 mL) and DCM (5 × 2 mL). Amino acids (0.2 mmol) were preactivated with OxymaPure (0.4 mmol), diisopropylcarbodiimide (DIC) (0.2 mmol) and DMF (1 mL), and added to the resin. Coupling time was 60 minutes for the first amino acid, and 40 minutes for all remaining amino acids at room temperature. After completion of the peptide chain and Fmoc deprotection, the resin was washed with DMF (5 × 2 mL) and DCM (5 × 2 mL), and the N-terminus was acetylated using 50/50 v/v pyridine and acetic anhydride (2 × 5 mins). After acetylation, the peptides were washed with DMF (5 × 2 mL) and DCM (5 × 2 mL). The peptides were cleaved from resin using a mixture of trifluoroacetic acid (TFA)/triisopropylsilane/H_2_O (90/5/5, 2 mL) at room temperature (2 hours). Following cleavage, TFA was evaporated with a stream of nitrogen gas, and subsequently the peptide was precipitated in diethyl ether (30 mL) and centrifuged (4500 rpm, 5 minutes). After centrifugation, diethyl ether was decanted, and the peptide was dissolved in 50% v/v MeCN in H_2_O (5 mL) and lyophilized. Impurities were removed using high-performance liquid chromatography at a gradient of 1% B/min. The final products were lyophilized and stored at −20 °C. Gla residues were incorporated using Fmoc-Gla(OtBu)_2_-OH using a modified coupling protocol; Fmoc-Gla(OtBu)_2_-OH (2 equivalents) was preactivated in situ with DIC (2 equivalents) and OxymaPure (4 equivalents) in 2 mL DMF for 1 minute, added to the growing peptide chain and coupled for 60 minutes at room temperature with continuous N_2_ bubbling.

### 2.2 Boc synthesis of peptides

4-Methylbenzhydrylamine resin (0.1 mmol) was swelled in DMF (60 minutes) in an solid phase peptide synthesis reactor. Amino acids were manually coupled using the following steps: in short, the resin was washed with DMF (2 × 5 mL), followed by addition of neat TFA for Boc deprotection (2 × 1 min). Amino acids (0.2 mmol) were simultaneously preactivated with O-(1H-6-chlorobenzotriazole-1-yl)-1,1,3,3-tetramethyluronium in DMF (0.5 M, 1 mL) and *i*-Pr_2_NEt (0.2 mL). Resin was washed with DMF (4 × 3 mL) and the preactivated amino acid was added to the resin and reacted for 20 minutes at room temperature with continuous nitrogen bubbling. Following peptide chain assembly, the final Boc group was removed, and the resin was washed with DMF (2 × 5 mL). Optionally, peptides were acetylated with acetic anhydride in pyridine (1:1). After acetylation, the peptide was washed again with DMF and DCM, and dried. *p*-Cresol (4% v/v) was added to the dry sample, and the sample was cleaved from solid support with anhydrous HF (1 hour, 0 °C). Gla residues were incorporated using Boc-Gla(OcHx)_2_-OH using a modified coupling protocol; resin was neutralized after TFA treatment using 5% *i*-Pr_2_Net in DMF (2 × 1 minute). Boc-Gla(OcHx)_2_-OH (2 equivalents) was preactivated in situ with DIC (2 equivalents) and OxymaPure (4 equivalents) in 2 mL DMF for 1 minute, added to the growing peptide chain and coupled for 60 minutes at room temperature with continuous N_2_ bubbling. After cleavage, the peptide was precipitated in diethyl ether and filtered. The peptides were redissolved in 50% v/v MeCN in H_2_O (25 mL). Impurities were removed using high-performance liquid chromatography at a gradient of 1% B/min. The final products were lyophilized and stored at −20 °C.

### 2.3 NMR titrations

NMR measurements were performed on a Bruker AVANCE III HD 700 MHz NMR spectrometer equipped with a TCI (^13^C, ^15^N, ^1^H) cryoprobe. All samples were measured at a temperature of 286 K. Peptide samples (10 mM or 2 mM for conantokin T) were prepared in 5-mm NMR tubes containing 600 μL MilliQ water with 2% (v/v) D_2_O for lock, DSS-d_6_ (3.2 mM) and EDTA (0.13 mM). DSS-d_6_ was used as the chemical shift reference (0 ppm), and a small excess EDTA (0.13 mM) was used to remove potential divalent metal impurities present in the peptide sample or solvent.

To measure the NMR pH titration curves, pH values per titration point were adjusted using small additions of milliliter aliquots of NaOH and HCl stock solutions (0.1 N, 0.2 N, and 1 N). pH values were measured before and after every NMR experiment with a Thermo Scientific Orion 3-star Benchtop pH meter (Fisher Scientific) at 21 °C, connected to a Thermo Scientific pH microelectrode (3 mm diameter). The pH meter was calibrated daily using standard calibration buffers (pH 4.01, 7.00, 10.01). To measure the Ca^2+^ titration curves, small aliquots of CaCl_2_ stock solution (100 mM and 1 M) were added stepwise to the prepared peptide samples at a constant pH (7.6). To assign all peptide resonances, several 2-dimensional (D) spectra were run at low pH and at regular intermediate pH intervals. The set of 2D spectra include Decoupling In the Presence of Scalar Interactions (DIPSI) (80 millseconds mixing time), Nuclear Overhauser Effect Spectroscopy (350–500 milliseconds mixing time), ^13^C-^1^H Heteronuclear Single Quantum Coherence (HSQC), ^13^C-HSQC-DIPSI (70 milliseconds mixing time), ^13^C-^1^H Constant Time-Heteronuclear Multiple Bond Correlation (CT-sHMBC) (using a 5- to 7.5-Hz transfer step optimized for detection of long-range proton to carbonyl connectivity’s of Glu and Gla residues and water presaturation during the relaxation delay).

One-dimensional ^1^H and ^13^C spectra were averaged >32 and 256 scans, respectively. HSQC spectra were run at standard for 8 scans for 512 increments each, the DIPSI was run at standard for 4 scans and 256 increments each, and the selective ^13^C-^1^H CT-sHMBC was run for 16 scans and 256 to 292 increments each, covering a spectral range of 18 ppm in the carbonyl region. Optimal delay times for magnetization transfer in the sHMBC experiment were based on the experimental determined 3J(CD-HB1/2) and 2J(CD-HG) coupling constants (4.05 and 6.6 Hz, respectively) extracted from the proton-coupled ^13^C spectrum of fully deprotonated ethylmalonate. For all spectra, the 90° pulse length for maximum sensitivity was calibrated at each individual pH point, and this value varied between 10 and 20 microseconds (depending on the varying salt concentration after pH adjustments). For most 1D and 2D spectra, a cosine-squared apodization function was used before zero filling. ^1^H spectra were recorded with 32 or 64 scans, and water suppressing was achieved using excitation sculpting with gradients. Specific pulse sequence schemes that were used Supplementary Table S4.

For all peptides at all pH points, chemical shift profiles of most Gla (Glu) ^13^C and ^1^H resonances could be reconstructed as a function of pH and Ca^2+^ concentration, derived from high resolution ^1^H and ^13^C (proton-decoupled) spectra with support of data assigned by 2D NMR spectra.

The acquisition software used was Topspin 3.6.5 (Bruker GmbH). Xcrvfit 5.0.3 [18] was used fit single and dual p*K*_a_ profiles for the Gla chemical shift pH titration curves, and GraphPad Prism 10.4.1. (Dotmatics) was used to fit all single sigmoid Ca^2+^-binding curves and to generate the graph plots.

Data were fitted using the following formula to determine the binding affinity for peptides 1 to 6:

(1) $${\Delta \delta }_{Obs}=\frac{[{Ca}^{2+}]+P+K_{D}-\sqrt{{\left([{Ca}^{2+}]+P+K_{D}\right)}^{2}-4*\left[{Ca}^{2+}\right]*P}}{2*P}*{\Delta \delta }_{\max}$$

where *P* represents the peptide concentration, [Ca^2+^] represents the Ca^2+^ concentration, Δδ_obs_ represents the observed change in chemical shift, Δδ_max_ represents the maximum change in chemical shift, and *K*_D_ represents the binding affinity.

To account for cooperativity in the case of conantokin T, data were fitted using the following formula:

(2) $${\Delta \delta }_{Obs}=\frac{\left({\Delta \delta }_{\max}*P\right)*{\left[{Ca}^{2+}\right]}^{n}}{K_{D}^{n}+{\left[{Ca}^{2+}\right]}^{n}}$$

where *P* represents the peptide concentration, [Ca^2+^] represents the Ca^2+^ concentration, Δδ_obs_ represents the observed change in chemical shift, Δδ_max_ represents the maximum change in chemical shift, *n* represents the Hill coefficient, and *K*_D_ represents the binding affinity.

### 2.4 CD spectroscopy of osteocalcin P15-N26 and conantokin T

CD spectra were recorded on a Chirascan v100 (Applied Photophysics) spectrometer operating at wavelengths between 190 and 260 nm, wavelength intervals of 0.5 nm, and half-bandwidths of 1 nm using a 1-mm quartz cuvette. The sample cell temperature was set to 20 °C. Before each sample measurement, a background measurement was performed without cuvette, followed by a blank measurement with experiment buffer.

Osteocalcin P15-N26 stock solutions (1 mg/mL in H_2_O with 100 μM EDTA) were diluted to 0.1 mg/mL in borate buffer (25 mM) with EDTA (100 μM) at pH 7.4 and mixed with 0%, 10%, 20%, or 30% trifluoroethanol (TFE). All samples were measured with and without CaCl_2_ (10 mM).

Conantokin T stock solutions (1 mg/mL in H_2_O with 100 μM EDTA) were diluted to 0.1 mg/mL in H_2_O and EDTA (100 μM). Conantokin T was measured at pH 2, 5, and 7.5 at 20 °C. At 20 °C and pH 7.5, 37.2 μM CaCl_2_, 186 μM CaCl_2_, and 372 μM CaCl_2_ were added to the conantokin T sample solution. Finally, at pH 7.5, a temperature titration was recorded from 20 °C to 80 °C at 5 °C increments.

Conversion to mean residue ellipticity was performed using the following formula:

(3) $$\theta =\frac{\;\text{Millidegrees}}{(\text{pathlength}\;\left(\text{cm}\right)*\left[\text{peptide}\right]\left(M\right)*\text{number}\;\text{of}\;\text{residues}}$$

To determine the percentage α-helicity, the following formula was used [19]:

(4) $$\%\;\text{helix}={\left[\theta \right]}_{\text{obs}}/-40,000*\left(n-4\right)/n$$

where ${\left[\theta \right]}_{obs}$ represents the observed ellipticity at 222 nm, and *n* represents the peptide chain length. Data were thereafter analyzed using GraphPad Prism version 10.4.1.

## 3 Results and Discussion

### 3.1 Determining Gla p*K*_a_ values in random coil model peptides

Calcium ion-binding properties of γ-carboxyglutamic acid likely depend on the protonation state of the amino acid. For this reason, we started with the determination of side-chain p*K*_a_ values of Gla within a peptide chain. In order to rule out effects of charged or bulky neighboring amino acids, model peptides were synthesized with Gla residues flanked by glycine residues and N-terminus and C-terminus were acetylated and amidated, respectively. We synthesized peptides with a single Glu residue (1), a single Gla residue (2), 2 Gla residues directly next to each other (3), or 2 Gla residues spaced by a glycine (4) (Supplementary Figure S1). The p*K*_a_ was determined by fitting chemical shift differences of Gla or Glu δ and/or γ carbons as a function of pH (Table 1 and Supplementary Tables S1, S5 and S6).

**Table 1:** Side-chain carboxylic acid *p*K_*a*_ values of glutamic acid- and γ-carboxyglutamic acid-containing model peptides for residues highlighted in bold.

Peptide	Sequence	No Ca^2+^		50 mM Ca^2+^		Δ 50 mM Ca^2+^-No Ca^2+^	
		p*K*_a1_	p*K*_a2_	p*K*_a1_	p*K*_a2_	p*K*_a1_	p*K*_a2_
**1**	Ac-GG**E**GG-CONH_2_	4.34	—	4.30	—	−0.03	—
**2**	Ac-GG**γ**GG-CONH_2_	2.47	4.99	2.54	4.58	0.07	−0.41
**3**	Ac-GG**γ**γGG-CONH_2_	2.66	5.05	2.52	4.51	−0.15	−0.54
	Ac-GGγ**γ**GG-CONH_2_	2.66	5.06	2.51	4.51	−0.15	−0.55
**4**	Ac-GG**γ**GγGG-CONH_2_	2.68	5.07	2.54	4.57	−0.13	−0.50
	Ac-GGγG**γ**GG-CONH_2_	2.70	5.08	2.56	4.57	−0.14	−0.51
**5**	Ac-PL**E**PRREVSELN-CONH_2_	4.26	—	4.25	—	−0.02	—
	Ac-PLEPRR**E**VSELN-CONH_2_	4.33	—	4.28	—	−0.05	—
	Ac-PLEPRREVS**E**LN-CONH_2_	4.19	—	4.20	—	0.01	—
**6**	Ac-PL**γ**PRRγVSγLN-CONH_2_	2.55	4.95	2.51	4.62	−0.04	−0.33
	Ac-PLγPRR**γ**VSγLN-CONH_2_	2.60	4.93	2.55	4.49	−0.05	−0.44
	Ac-PLγPRRγVS**γ**LN-CONH_2_	2.66	5.07	2.54	4.56	−0.12	−0.51

Gla residues are denoted as γ. p*K*_a_ values were determined based on δ-carbon and γ-carbon chemical shift differences, average values are reported. Peptide concentration was 10 mM in all experiments.

**Figure 3:**
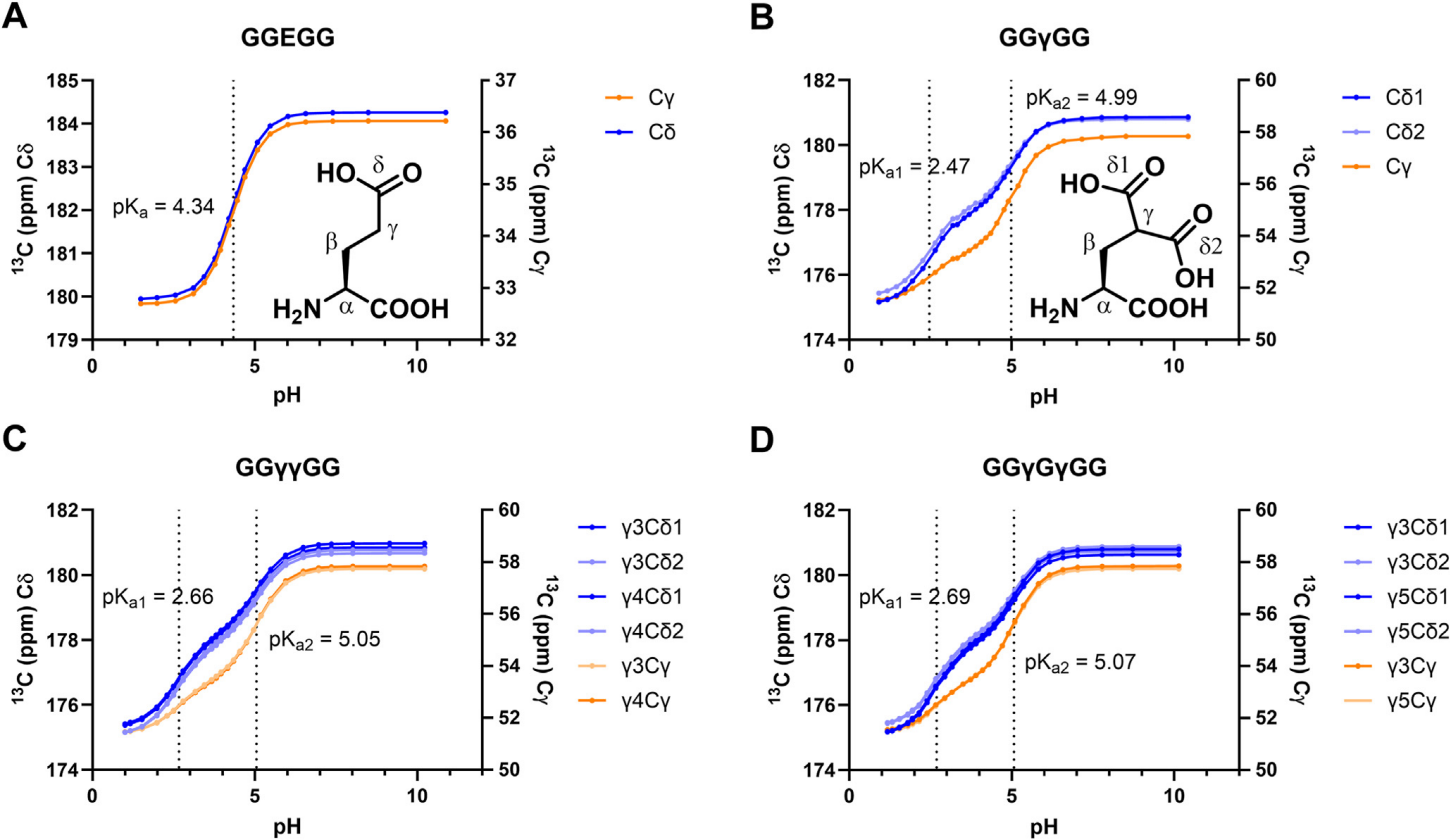
Chemical shift of γ-carboxyglutamic acid (Gla) δ (blue) and γ (orange) carbons as a function of pH for (A) GGEGG (B) GGγGG (C) GGγγG and (D) GGγGγGG. Numbering of δ carbons is arbitrary. Inflection points are indicated with dotted lines. Insets in (A) and (B) show chemical structure including numbering of carbon atoms of Glu and Gla, respectively. ^13^C data points were averaged over 256 scans.

Glu-containing peptide 1 displayed a typical sigmoid curve (Figure 3A), with a single inflection point and an observed p*K*_a_ of 4.34, which is consistent with previously reported values for glutamic acid (4.34) [20]. Similar to other dicarboxylic acids such as malonic or oxalic acid, Gla-containing peptides were expected to have 2 unique p*K*_a_ values [21]. Moreover, previous research had indicated that titration curves for γ-carboxyglutamic acid appeared to be biphasic [22].

As expected, titration curves of peptides 2 to 4 showed 2 distinct inflection points and thus 2 p*K*_a_ values for each of the Gla residues present (Table 1 and Figure 3B–D). While absolute chemical shift differences were larger for δ carbons than for those γ carbons and curves may appear different, fitted p*K*_a_ values were similar (Supplementary Table S1). p*K*_a_ values for peptides 2 to 4 ranged from 2.47 to 2.70 for p*K*_a1_ and 4.99 to 5.08 for p*K*_a2_. We did not observe significant effects of directly adjacent Gla residues or those in spatial proximity, although p*K*_a1_ is slightly lower in peptide 2 with a single Gla residue compared with peptides 3 and 4 with 2 Gla residues. We next moved away from a model system to a more physiological peptide sequence to investigate whether the protonation state could be influenced by other amino acids, such as positively charged arginines, bulky hydrophobic amino acids such as leucine and valine, or other Gla residues. Two peptides derived from human osteocalcin (P15-N26 [PLγPRRγVSγLN]) with 3 γ-carboxyglutamic acid residues in both uncarboxylated (5) and carboxylated form (6) were therefore synthesized. The native cysteine on position 23 was replaced with serine to avoid disulfide formation. Uncarboxylated glutamic acid residues in 5 showed p*K*_a_ values comparable with those found in model peptide 1. Similarly to model peptides 2 to 4, peptide 6 showed 2 inflection points for each of the Gla residues present (Supplementary Figure S2). For all 3 Gla residues present, both p*K*_a_ values of 6 were within range of those found in peptides 2 to 4 (Table 1). We therefore conclude that in isolated random coil peptide structures, p*K*_a_ values of Gla are constant regardless of neighboring amino acids or number of Gla residues.

### 3.2 Calcium ion binding leads to a more stable charged state of Gla residues

**Figure 4:**
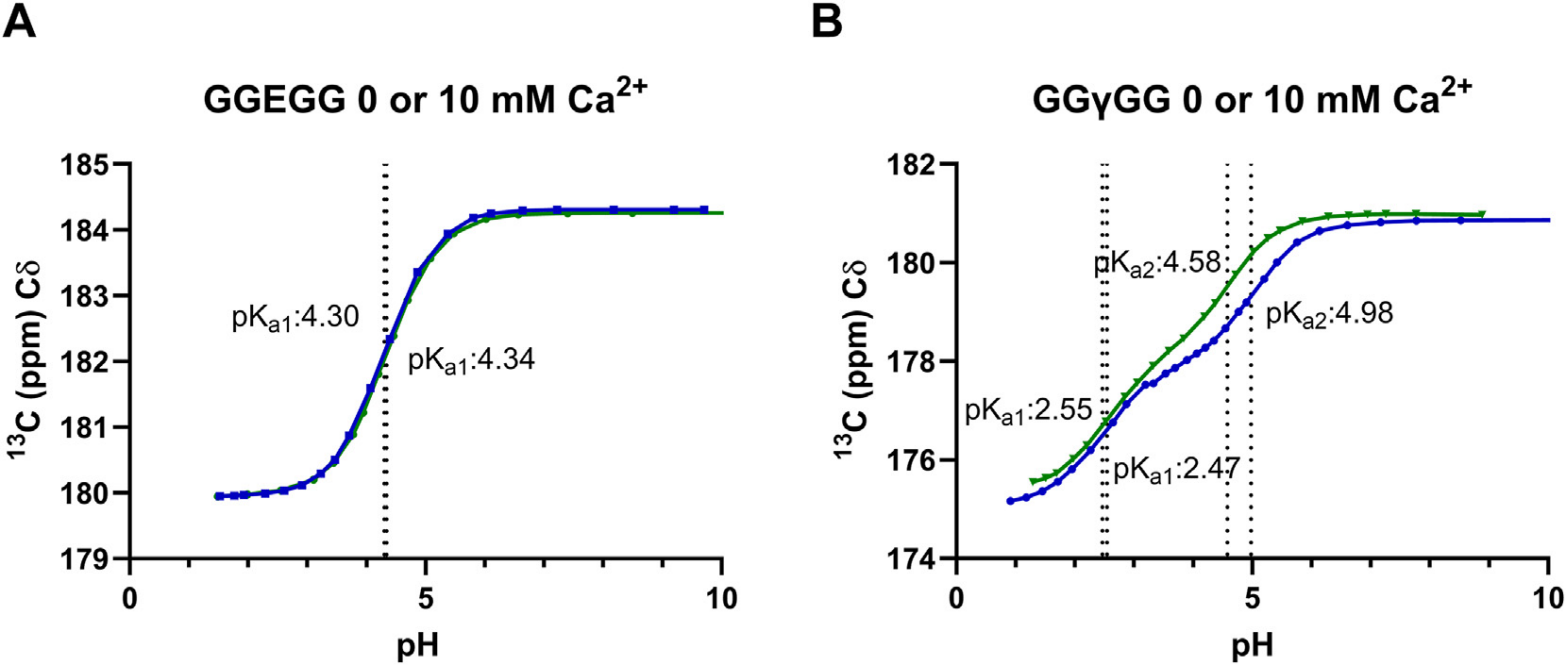
Chemical shift of γ-carboxyglutamic acid (Gla) δ carbons as a function of pH in absence (blue) and presence (green) of a 5-fold excess of calcium ions for (A) GGEGG and (B) GGγGG. Inflection points are indicated with vertical dotted lines. ^13^C data points were averaged over 256 scans.

Plasma concentrations of calcium range from 2.0 to 2.6 mM; Gla residues present in coagulation factors are thus always exposed to calcium ions. To investigate whether Ca^2+^ contributes to an energetically favorable environment for charge stabilization of the Gla carboxyl side chains, pH titrations were repeated in presence of a 5-fold excess of CaCl_2_ (10 mM peptide and 50 mM CaCl_2_). As expected, addition of Ca^2+^ had a negligible effect on the p*K*_a_ values of uncarboxylated peptides 1 and 5 (Figure 4A, Table 1, and Supplementary Figure S3). Carboxylated peptides 2 to 4 and 6 showed a minor shift (average, −0.09) for the first inflection point as a result of adding Ca^2+^. However, the second inflection point showed a larger shift to lower pH, indicating a reduction in p*K*_a_ (average, −0.47) (Figure 4B, Table 1, Supplementary Table S2 and Supplementary Figures S3 and S4). This may be explained by Ca^2+^ bridges being formed between Gla carboxylic acid side chains, stabilizing their charge, making the second carboxylic acid easier to deprotonate [23].

### 3.3 Calcium ion affinity

Having determined the protonation state of γ-carboxyglutamic acid in presence and absence of calcium, we next focused on the affinity of the individual Gla residues for calcium. While Ca^2+^ binding by Gla-containing proteins has been known since the discovery of the carboxylated amino acid [24,25], binding affinities of individual Gla residues for calcium ions are incomplete. In addition to calcium, other ions (Mg^2+^ and Mn^2+^) have been implicated to bind Gla or Gla domains [10,26]. Previous studies have observed high affinity for insoluble calcium salts [27] and defined 2 types (tight and lower affinity) of metal ion binding in coagulation factor Gla domains. These studies, however, focused on the isolated Gla amino acid [28], prothrombin Gla domain [29], or protein C Gla domain [14,15]. We were therefore interested in calcium binding affinity of isolated and grouped Gla residues in a protein environment and in a general and systematic manner. The *K*_D_ was determined by measuring chemical shift of Gla or Glu δ and/or γ carbons as a function of calcium ion concentration (Table 2). Ca^2+^ titrations were performed at physiological pH (7.4) where all carboxylic acid residues were deprotonated based on their previously determined p*K*_a_ values. In addition to calcium titrations, we performed titrations of ethylmalonate with CaCl_2_, MgCl_2_, ZnCl_2_, and LuCl_3_. However, precipitation of salt (in case of Zn^2+^ and Lu^3+^) or a determined general lower affinity of Mg^2+^ compared with Ca^2+^, led to us not pursuing these metals further in a systematic study to quantify metal-binding effects in Gla peptides. Moreover, negatively charged membranes may influence calcium binding by Gla-containing peptides and proteins and will be subject of future investigations [11,16].

**Table 2:** Calcium ion affinity of Gla-containing model peptides.

Peptide	Sequence	*K*_D_ (mM)
**1**	Ac-GG**E**GG-CONH_2_	>100 mM
**2**	Ac-GG**γ**GG-CONH_2_	14.3
**3**	Ac-GG**γγ**GG-CONH_2_	16.7 (Gla 3) 17.5 (Gla 4)
**4**	Ac-GG**γ**G**γ**GG-CONH_2_	18.0 (Gla 3) 19.0 (Gla 5)
**5**	Ac-PL**E**PRR**E**VS**E**LN-CONH_2_	>100 mM (Glu 3) >100 mM (Glu 7) >100 mM (Glu 10)
**6**	Ac-PL**γ**PRR**γ**VS**γ**LN-CONH_2_	29.4 (Gla 3) 8.3 (Gla 7) 5.9 (Gla 10)
**7**	NH_2_-GE**γγ**YQKML**γ**NLR**γ**AEVKKNA-CONH_2_	9.7 (Gla 3) 40.8 (Gla 4) 0.7 (Gla 10) 0.6 (Gla 14)

As expected, peptide 1, which contains a single glutamic acid residue, showed no significant affinity for calcium ions (Table 2 and Supplementary Figure S5). In contrast, Gla-containing peptide 2 showed binding to calcium ions with a moderate affinity of 14.3 mM. As previously mentioned, crystal structures of Gla domains show a ratio of 2 Gla residues per calcium ion. Gla residues in peptides 3 and 4 were therefore expected to show increased binding affinity. However, no change in affinity (*K*_D_, 17.1 and 18.5 mM) was observed for both peptides. Theoretically, multiple peptides may be able to interact with a single calcium ion. NMR experiments, however, were performed at high peptide concentrations (10 mM), with calcium concentrations exceeding peptide concentrations by 20-fold and dilution experiments did not show significant changes in NMR spectra. We therefore believe this is not the case in our experiments. We hypothesized that the lack of affinity increase in peptides with multiple Gla residues may be caused by incorrect spatial positioning of the carboxyl groups, thus prohibiting cooperative binding. Osteocalcin-derived peptides 5 and 6 form an α-helix when present in the native, full-length protein and present Gla residues on one side of this helix, optimally spaced for cooperative binding of calcium ions [12]. While uncarboxylated Glu residues in peptide 5 showed no affinity for calcium ions (Supplementary Figure S6), binding affinity increased slightly for 2 of 3 Gla residues present in peptide 6 (Table 2 and Supplementary Figure S6). NMR analysis of peptides 5 and 6, however, did not show evidence of an α-helical fold of this fragment, which may once more have led to incorrect positioning of carboxyl groups for cooperative calcium ion binding.

### 3.4 Secondary structure is required for cooperative calcium ion binding

**Figure 5:**
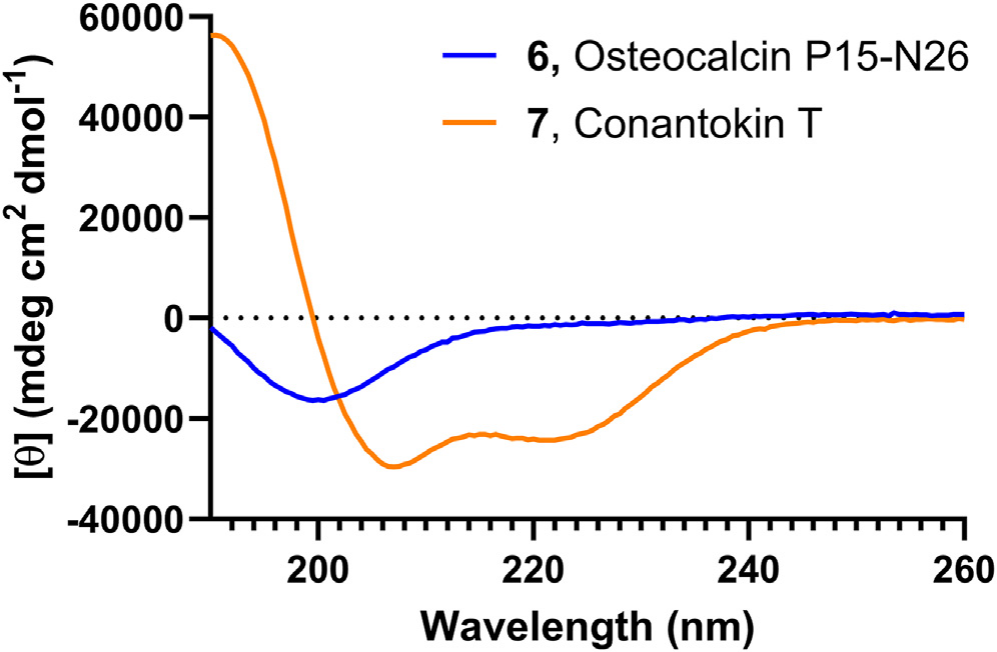
Circular dichroism of 6 osteocalcin P15-N26 (blue) and **7** conantokin T (orange), both at 0.1 mg/mL in H_2_O.

To confirm the absence of an α-helical fold and thus suboptimal positioning of Gla residues in osteocalcin-derived peptide 6, CD spectroscopy was performed. CD analysis showed 6 was present in random coil in aqueous solution without additives (Figure 5); α-helical content was quantified to be 5%. Helicity could, however, dose dependently be induced by addition of TFE and 10 mM CaCl_2_ to a maximum of 20% (Supplementary Figure S7 and Supplementary Table S3). While addition of TFE led to increased secondary structure and might have positioned Gla residues better for cooperative calcium ion binding, this proved incompatible with the chosen NMR analysis. For this reason, we decided to use a Gla-containing peptide, which readily forms an α-helix without the requirement of Ca^2+^ or TFE to investigate how secondary structure can aid cooperative Ca^2+^ binding. conantokin T (**7**), a 21-residue long peptide containing 4 Gla residues found in the venom of marine snails with high helical content was used for this purpose [13,19]. CD analysis confirmed that conantokin T was 70% α-helix at pH 7.5 (Figure 5 and Supplementary Table S3). Helicity could be increased by addition of calcium to a maximum of 96% at 10-fold excess of CaCl_2_. Helicity was furthermore shown to be dependent on both pH and temperature, as increased temperature or lower pH showed a decrease in helicity (Supplementary Figure S7 and Supplementary Table S3). Interestingly, Gla residues in conantokin T are positioned both next to each other (Gla3 and Gla4) and in *i*, *i* + 4 fashion (Gla10 and Gla14) (Figure 1C). In an α-helical fold, this results in Gla3 and Gla4 pointing away from each other, while Gla10 and Gla14 are presented on the same side of the helix and optimally spaced for cooperative calcium ion binding.

A Ca^2+^ titration (0–35 mM) was performed to determine affinity of individual Gla residues in conantokin T (concentration 2 mM) for calcium ions. In agreement with CD analysis, NMR showed α-helical patterns without the addition of Ca^2+^ or TFE; Cα shifted downfield and the Hα shift was lower than the POTENCI predicted random coil chemical shifts of conantokin T (Supplementary Table S7) [30]. Furthermore, with addition of calcium ions, helicity gradually increased and became saturated at 5-fold excess of Ca^2+^.

**Figure 6:**
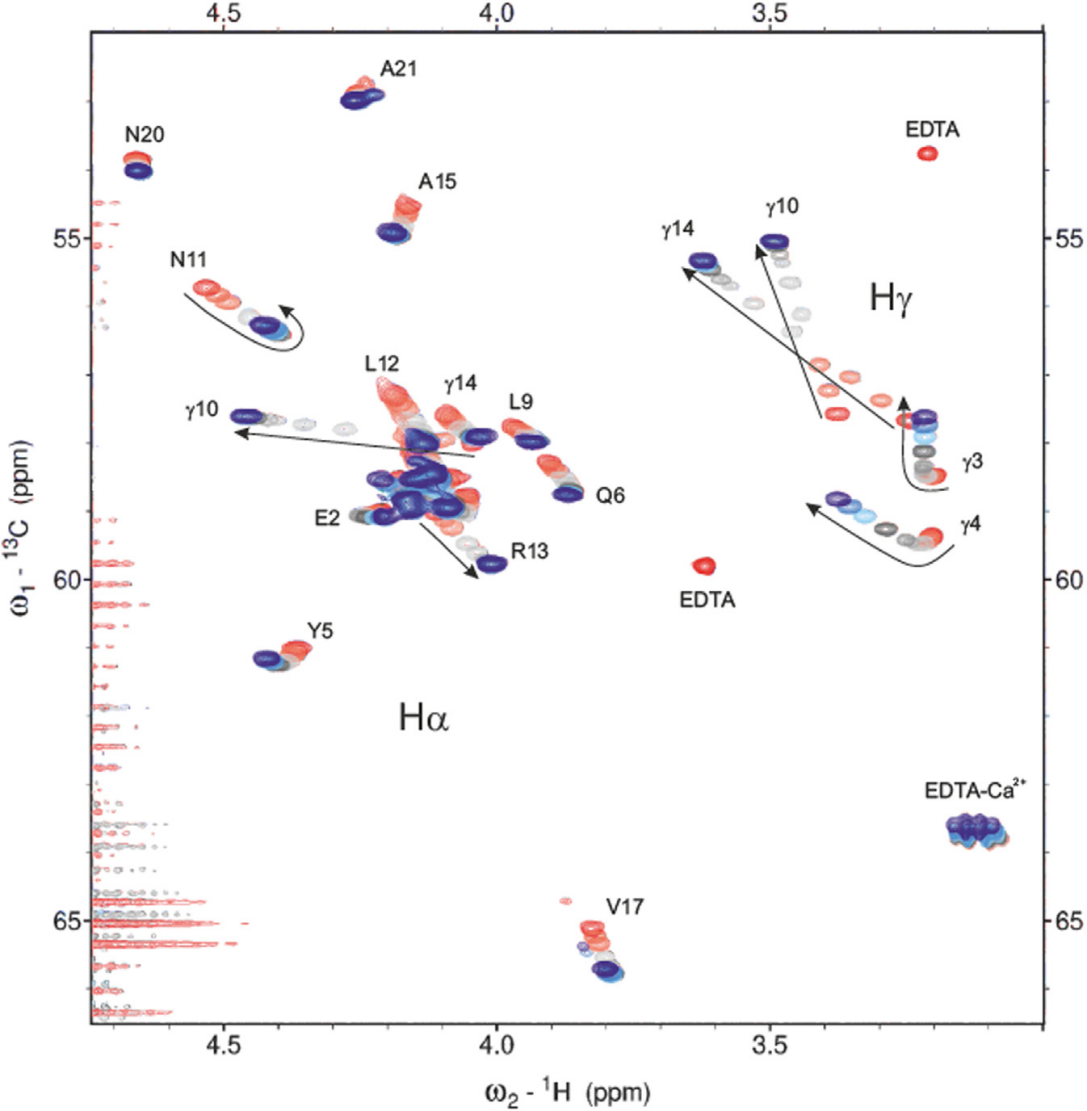
Heteronuclear single-quantum coherence ^13^C-^1^H HSQC stack plot of the Ca/Hα region (∼3.7–4.7 ppm ^1^H) and γ-carboxyglutamic acid (Gla) Ca/Hγ regions (∼3.1–3.7 ppm ^1^H). Red peaks represent the apo conantokin T form (unbound EDTA peaks are visible), and blue peaks represent 2 mM conantokin T with 35 mM of Ca^2+^ added. To ensure a calcium ion-free apo conantokin T state at the start of the titration a slight excess of EDTA was initially added to reference conantokin T (red EDTA peaks); peaks shift to new positions upon Ca^2+^ binding (blue EDTA-Ca^2+^).

**Figure 7:**
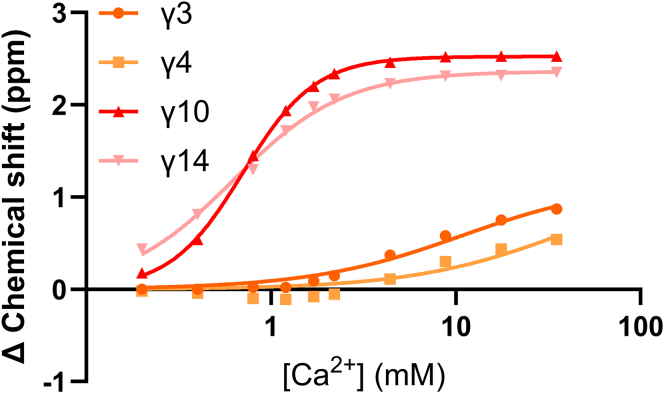
Ca^2+^ titration of conantokin T. Change in carbon γ chemical shifts was measured for each of the γ-carboxyglutamic acid(Gla) residues. Data points were fitted using quadratic (Gla3 and Gla4) or cooperative (Gla10 and Gla14) binding models.

In contrast to Gla-containing peptides 2 to 4 and 6, the addition of Ca^2+^ to conantokin T showed 2 sets of binding modes (Figure 6). Where Gla residues 3 and 4 showed similar binding affinities (9.7 and 40.8 mM, respectively) as those found in earlier analyzed random coil model peptides, Gla residues 10 and 14 had higher affinities (0.7 and 0.6 mM, respectively) for calcium ions (Figure 7). Moreover, binding curves for Gla10 and Gla14 could not be fitted with a quadratic binding solution but could be fitted with a cooperative binding model. Derived Hill coefficients for this fit were *n* = 2.21 and *n* = 1.45, respectively, indicating positive cooperativity of binding. We therefore believe this increase in binding affinity is the result of cooperative calcium ion binding by the 2 Gla residues present on the same side of the α-helix formed by conantokin T. In addition, delta Cγ and Hγ chemical shifts of Gla10 and Gla14 were much larger than those found earlier. This may be the result of a combination of calcium ion binding as well as stabilization of the α-helical fold of conantokin T. The individual fitted *K*_D_ values were in range with those found for the full protein in previous studies [31]. Moreover, individual binding affinities found are in range of those found for individual Gla residues in human protein C [15].

## 4 Conclusion

Gla domains play an essential role in coagulation and mineralization through their calcium ion-binding properties. However, biophysical properties such as p*K*_a_ and calcium ion affinity of individual Gla residues are not fully known. Using a NMR approach, we could determine the protonation state and calcium ion-binding affinity of Gla residues in different model peptides in random coil and α-helical structure. We have shown that the protonation state of γ-carboxyglutamic acid is dependent on calcium ion binding as this leads to a more stable charged state of Gla. Carboxylic acid groups present on Gla residues have 2 different p*K*_a_ values of 2.62 ± 0.07 and 5.02 ± 0.05. In presence of calcium ions, p*K*_a_ values drop to 2.54 ± 0.02 and 4.55 ± 0.04, respectively. Moreover, we determined the calcium ion-binding affinity of individual Gla residues in a peptide chain. Based on these results, we concluded that a single Gla residue is able to bind calcium with an affinity of approximately 15 mM. However, we could show that calcium ion binding is cooperative and affinity increased to 0.7 mM, when 2 Gla residues are in close proximity with correct spatial orientation. This illustrates the fact that under physiological calcium concentration conditions (blood plasma, 2 mM), it takes 2 Gla residues to internally tether a calcium ion. Finally, our experimentally determined biophysical properties can now be used for prediction and molecular modeling of Gla domains with unknown structure or properties.
