# Supplementary material for: Calcium binding by γ-carboxyglutamic acid: it takes two to tether
Source: Res Pract Thromb Haemost. 2025 Jun 27;9(5):102964. doi: 10.1016/j.rpth.2025.102964 (PMC12312033; doi:10.1016/j.rpth.2025.102964)
Supplement: Supplementary Material [file mmc1.docx]

Supplemental information

Calcium binding by γ-carboxy glutamic acid: it takes two to tether

Hans Ippel, Sem J Peijnenborgh, Tilman M Hackeng, Stijn M Agten.

CARIM, Biochemistry, Maastricht, Netherlands

[s.agten@maastrichtuniversity.nl](mailto:s.agten@maastrichtuniversity.nl)

ORCID:

HI: 0000-0001-6589-6678

SJP: 0009-0009-2800-3768

TMH: 0000-0003-0142-0843

SMA: 0000-0002-5973-272X

|  |  |
| --- | --- |
|  |  |
|  |  |
|  |  |

Figure S1: MS spectra of synthesized peptides **1-7**, **A**: Ac-GGEGG-CONH_2_ **B**: Ac-GGγGG-CONH_2_ **C**: Ac-GGγγGG-CONH_2_ **D**: Ac-GGγGγGG-CONH_2_ **E**: Ac-PLEPRREVSELN-CONH_2_ **F**: Ac-PLγPRRγVSγLN-CONH_2_, **G**: NH_2_-GEγγYQKMLγNLRγAEVKKNA-CONH_2_ (Conantokin T).

Figure S2: NMR pH titration curves of osteocalcin (P15-N26) derived peptides , **A**: Osteocalcin P15-N26 Glu **B**: Osteocalcin P15-N26 Gla δ refers to the side chain carboxyl group(s) of Gla or Glu. γ refers to Gla, and E refers to Glu. *In the case of Gla containing peptides, the number for the side-chain δ carboxyl carbons is arbitrary (δ1/ δ2), identifying δ carbons was not performed. The δ carbon with higher chemical shift (ppm) was consistently referred to as δ1, and the δ carbon with lower chemical shift (ppm) was referred to as δ2. Dotted lines represent fitted pKa values.

Figure S3: NMR pH titrations of Gla and Glu containing peptides in presence of 50 mM Ca^2+^. **A**: Ac-GGEGG-CONH_2_, **B**: Ac-GGγGG-CONH_2_, **C**: Ac-GGγγGG-CONH_2_ **D**: Ac-GGγGγGG-CONH_2_. δ refers to the side chain carboxyl group(s) of Gla or Glu. γ refers to Gla, and E refers to Glu. *In the case of Gla containing peptides, the number for the side-chain δ carboxyl carbons is arbitrary (δ1/ δ2), since knowing which delta carbon was which was not measured. The δ carbon with higher chemical shift (ppm) was consistently referred to as δ1, and the δ carbon with lower chemical shift (ppm) was referred to as δ2.

Figure S4: NMR pH titration curves of osteocalcin (P15-N26) derived peptides in presence of 50 mM Ca^2+^ , **A**: Osteocalcin P15-N26 Glu **B**: Osteocalcin P15-N26 Gla δ refers to the side chain carboxyl group(s) of Gla or Glu. γ refers to Gla, and E refers to Glu. *In the case of Gla containing peptides, the number for the side-chain δ carboxyl carbons is arbitrary (δ1/ δ2), identifying δ carbons was not performed. The δ carbon with higher chemical shift (ppm) was consistently referred to as δ1, and the δ carbon with lower chemical shift (ppm) was referred to as δ2. Dotted lines represent calculated pKa values.

Figure S5: 0-200 mM Ca^2+^ titrations of Glycine flanked peptides, fitted with equation I (vide supra). **A**: Ac-GGEGG-CONH_2_, **B**: Ac-GGγGG-CONH_2_, **C**: Ac-GGγγGG-CONH_2_ **D**: Ac-GGγGγGG-CONH_2_.

Figure S6: 0-200 mM Ca^2+^ titrations of osteocalcin (P15-N26) derived peptides and conantokin T. Curves were fitted with equation I for both Osteocalcin peptides and γ3+γ4 of conantokin T. Curves of γ10 and γ14 of conantokin T were fitted with equation II (vide supra) **A**: Osteocalcin P15-N26 Glu **B**: Osteocalcin P15-N26 Gla **C**:Conantokin T.

Figure S7: Circular dichroism spectra of purified Osteocalcin P15-N26 Gla (A,B) (0.1 mg/mL) and Conantokin T (C,D) (37.2 μM) in water with EDTA (90 μM). A: CD of Osteocalcin P15-N26 Glu in borate buffer, with 10%, 20% or 30% Trifluoroethanol (TFE). B: CD spectrum of Osteocalcin P15-N26 Gla in borate buffer, with 10%, 20% or 30% TFE and 10 mM Ca^2+^. C: ConT with 37.2 μM Ca^2+^, 186 μM Ca^2+^ and 372 μM Ca^2+^ at 20 °C, pH 7.5 from 190 nm to 260 nm. D: CD spectrum of ConT at changing temperatures from 20°C to 80°C at 5°C increments measured at wavelengths 190 nm to 260 nm.

Table S1: Calculated pKa values of γ-carboxyglutamic acid in random coil and (partially) structured peptides. ^a^pKa values are an average of δ1 and δ2. ^b^due to spectral overlap, chemical shifts for γ carbons could not be followed over the entire pH range.

|  | | γ carbon |  |  | δ carbons^a^ | |  | average |  |
| --- | --- | --- | --- | --- | --- | --- | --- | --- | --- |
| **peptide** | **sequence** | **pKa1** | **pKa2** |  | **pKa1** | **pKa2** |  | **pKa1** | **pKa2** |
| 1 | Ac-GG**E**GG-CONH_2_ | 4.34 | N/A |  | 4.34 | N/A |  | 4.34 | N/A |
| 2 | Ac-GG**γ**GG-CONH_2_ | 2.48 | 5.01 |  | 2.47 | 4.98 |  | 2.47 | 4.99 |
| 3a | Ac-GG**γ**γGG-CONH_2_ | 2.70 | 5.06 |  | 2.63 | 5.04 |  | 2.66 | 5.05 |
| 3b | Ac-GGγ**γ**GG-CONH_2_ | 2.68 | 5.05 |  | 2.64 | 5.06 |  | 2.66 | 5.06 |
| 4a | Ac-GG**γ**GγGG-CONH_2_ | 2.69 | 5.07 |  | 2.67 | 5.07 |  | 2.68 | 5.07 |
| 4b | Ac-GGγG**γ**GG-CONH_2_ | 2.72 | 5.08 |  | 2.68 | 5.08 |  | 2.70 | 5.08 |
| 5a | Ac-PL**E**PRREVCELN-CONH_2_ | ND^b^ | N/A |  | 4.26 | N/A |  | 4.26 | N/A |
| 5b | Ac-PLEPRR**E**VCELN-CONH_2_ | ND^b^ | N/A |  | 4.33 | N/A |  | 4.33 | N/A |
| 5c | Ac-PLEPRREVC**E**LN-CONH_2_ | ND^b^ | N/A |  | 4.19 | N/A |  | 4.19 | N/A |
| 6a | Ac-PL**γ**PRRγVSγLN-CONH_2_ | ND^b^ | ND^b^ |  | 2.55 | 4.95 |  | 2.55 | 4.95 |
| 6b | Ac-PLγPRR**γ**VSγLN-CONH_2_ | ND^b^ | ND^b^ |  | 2.60 | 4.93 |  | 2.60 | 4.93 |
| 6c | Ac-PLγPRRγVS**γ**LN-CONH_2_ | ND^b^ | ND^b^ |  | 2.66 | 5.07 |  | 2.66 | 5.07 |

Table S2: Calculated pKa values of γ-carboxyglutamic acid in random coil and (partially) structured peptides in presence of 5 equivalents of CaCl_2_. ^a^pKa values are an average of δ1 and δ2. ^b^due to spectral overlap, chemical shifts for γ carbons could not be followed over the entire pH range.

|  | | γ carbon |  |  | δ carbons^a^ |  |  | average |  |
| --- | --- | --- | --- | --- | --- | --- | --- | --- | --- |
| **peptide** | **sequence** | **pKa1** | **pKa2** |  | **pKa1** | **pKa2** |  | **pKa1** | **pKa2** |
| 1 | Ac-GG**E**GG-CONH_2_ | 4.30 | N/A |  | 4.30 | N/A |  | 4.30 | N/A |
| 2 | Ac-GG**γ**GG-CONH_2_ | 2.53 | 4.57 |  | 2.55 | 4.58 |  | 2.54 | 4.58 |
| 3a | Ac-GG**γ**γGG-CONH_2_ | 2.54 | 4.52 |  | 2.50 | 4.50 |  | 2.52 | 4.51 |
| 3b | Ac-GGγ**γ**GG-CONH_2_ | 2.52 | 4.51 |  | 2.50 | 4.51 |  | 2.51 | 4.51 |
| 4a | Ac-GG**γ**GγGG-CONH_2_ | 2.57 | 4.57 |  | 2.52 | 4.58 |  | 2.54 | 4.57 |
| 4b | Ac-GGγG**γ**GG-CONH_2_ | 2.58 | 4.56 |  | 2.53 | 4.57 |  | 2.56 | 4.57 |
| 5a | Ac-PL**E**PRREVCELN-CONH_2_ | ND^b^ | N/A |  | 4.25 | N/A |  | 4.25 | N/A |
| 5b | Ac-PLEPRR**E**VCELN-CONH_2_ | ND^b^ | N/A |  | 4.28 | N/A |  | 4.28 | N/A |
| 5c | Ac-PLEPRREVC**E**LN-CONH_2_ | ND^b^ | N/A |  | 4.20 | N/A |  | 4.20 | N/A |
| 6a | Ac-PL**γ**PRRγVSγLN-CONH_2_ | ND^b^ | ND^b^ |  | 2.51 | 4.62 |  | 2.51 | 4.62 |
| 6b | Ac-PLγPRR**γ**VSγLN-CONH_2_ | ND^b^ | ND^b^ |  | 2.55 | 4.49 |  | 2.55 | 4.49 |
| 6c | Ac-PLγPRRγVS**γ**LN-CONH_2_ | ND^b^ | ND^b^ |  | 2.54 | 4.56 |  | 2.54 | 4.56 |

Table S3: Circular Dichroism of Osteocalcin P15-N26 Gla and Conantokin T. *[θ] refers to mdeg×cm^2^/dmol

| **Osteocalcin** **P15-N26 Gla** | **Molar Ellipticity at 222 nm [θ]*** | **% Helix** |
| --- | --- | --- |
| Apo, no TFE | -1120.2 | 4.2 |
| 10% TFE, 10 mM Ca^2+^ | -1698.1 | 6.4 |
| 20% TFE, 10 mM Ca^2+^ | -3588.5 | 13.5 |
| 30% TFE, 10 mM Ca^2+^ | -5533.6 | 20.8 |
|  |  |  |
| **Conantokin T** |  |  |
| 20°C, pH 7.5, 0 μM Ca^2+^ | -22993.1 | 71.0 |
| 20°C, pH 7.5, 37.2 μM Ca^2+^ | -28652.9 | 88.5 |
| 20°C, pH 7.5, 186 μM Ca^2+^ | -30840.6 | 95.2 |
| 20°C pH 7.5, 372 μM Ca^2+^ | -30980.9 | 95.7 |
|  |  |  |
| 20°C pH 7.5, 372 μM Ca^2+^ | -30980.9 | 95.7 |
| 25°C pH 7.5, 372 μM Ca^2+^ | -29965.3 | 92.5 |
| 30°C pH 7.5, 372 μM Ca^2+^ | -28292.9 | 87.4 |
| 35°C pH 7.5, 372 μM Ca^2+^ | -26855.6 | 82.9 |
| 40°C, pH 7.5, 372 μM Ca^2+^ | -25667.6 | 79.3 |
| 45°C, pH 7.5, 372 μM Ca^2+^ | -24422.4 | 75.4 |
| 50°C, pH 7.5, 372 μM Ca^2+^ | -22980.5 | 71.0 |
| 55°C, pH 7.5, 372 μM Ca^2+^ | -21704.6 | 67.0 |
| 60°C, pH 7.5, 372 μM Ca^2+^ | -20563.8 | 63.5 |
| 65°C, pH 7.5, 372 μM Ca^2+^ | -19421.1 | 60.0 |
| 70°C, pH 7.5, 372 μM Ca^2+^ | -18384.6 | 56.8 |
| 75°C, pH 7.5, 372 μM Ca^2+^ | -17460.1 | 53.9 |
| 80°C, pH 7.5, 372 μM Ca^2+^ | -16775.1 | 51.8 |
|  |  |  |
| 20°C pH 5, 372 μM Ca^2+^ | -28840.8 | 89.1 |
| 25°C pH 5, 372 μM Ca^2+^ | -27343.6 | 84.4 |
| 30°C pH 5, 372 μM Ca^2+^ | -25580.6 | 79.0 |
| 35°C pH 5, 372 μM Ca^2+^ | -24044.4 | 74.3 |
| 40°C, pH 5, 372 μM Ca^2+^ | -22450.8 | 69.3 |
| 45°C, pH 5, 372 μM Ca^2+^ | -20797.5 | 64.2 |
| 50°C, pH 5, 372 μM Ca^2+^ | -19434.9 | 60.0 |
| 55°C, pH 5, 372 μM Ca^2+^ | -18078.3 | 55.8 |
| 60°C, pH 5, 372 μM Ca^2+^ | -17250.5 | 53.3 |
| 65°C, pH 5, 372 μM Ca^2+^ | -16189.7 | 50.0 |
| 70°C, pH 5, 372 μM Ca^2+^ | -15144.2 | 46.8 |
| 75°C, pH 5, 372 μM Ca^2+^ | -14388.5 | 44.4 |
| 80°C, pH 5, 372 μM Ca^2+^ | -13164.3 | 40.7 |
|  |  |  |
| 20°C pH 2, 372 μM Ca^2+^ | -26037.5 | 80.4 |
| 25°C pH 2, 372 μM Ca^2+^ | -24860.1 | 76.8 |
| 30°C pH 2, 372 μM Ca^2+^ | -23345.9 | 72.1 |
| 35°C pH 2, 372 μM Ca^2+^ | -21986.1 | 67.9 |
| 40°C, pH 2, 372 μM Ca^2+^ | -20535 | 63.4 |
| 45°C, pH 2, 372 μM Ca^2+^ | -19399.1 | 59.9 |
| 50°C, pH 2, 372 μM Ca^2+^ | -18344.9 | 56.7 |
| 55°C, pH 2, 372 μM Ca^2+^ | -17006.3 | 52.5 |
| 60°C, pH 2, 372 μM Ca^2+^ | -16193 | 50.0 |
| 65°C, pH 2, 372 μM Ca^2+^ | -15272.7 | 47.2 |
| 70°C, pH 2, 372 μM Ca^2+^ | -14199 | 43.8 |
| 75°C, pH 2, 372 μM Ca^2+^ | -13485.7 | 41.6 |
| 80°C, pH 2, 372 μM Ca^2+^ | -12580.7 | 38.9 |

Table S4: Nuclear magnetic resonance pulse schemes.* All pulse schemes have been adapted from standard sequences in the Bruker library other than shmbcctetgpprl2nd.hi, which was a custom pulse sequence. Descriptions are

| Pulse Scheme | Description |
| --- | --- |
| zgesgp | 1D excitation sculpting using 180 degree water-selective pulses. |
| zgpg | ^13^C 1D Sequence with power-gated decoupling. |
| dipsi2esgpph | Phase-sensitive 2D TOCSY with excitation sculpting (180 water selective pulse es element) using DIPSI-2. |
| noesyesfbgpph | Phase-sensitive 2D NOESY with excitation sculpting (180 water selective pulse ES element) using water flip-back. |
| hsqcetgpsisp2 | Phase-sensitive ge-2D HSQC using preservation of equivalent pathways (PEP) and adiabatic pulses for inversion with gradients in back-inept. |
| shmbcctetgpprl2nd.hi* | Phase-sensitive band selective constant-time-HMBC using echo/antiecho gradient selection. Absolute magnitude mode calculation processing. With additional water presaturation step during relaxation delay. |
| roesyesgpph | Phase-sensitive 2D ROESY with excitation sculpting (180 water selective pulse ES element). |
| hsqcetfpf3gpsi2 | Phase-sensitive gradient enhanced 2D ^1^H ^15^N HSQC using PEP with gradient in back-inept. |
| hsqcdietgpsisp.2 | Phase-sensitive gradient enhanced 2D HSQC-TOCSY with DIPSI-2 using PEP and adiabatic inversion and refocusing pulses. |

Table S5: ^1^H chemical shifts (ppm) of Gla and Glu containing model peptides at indicated pH values. GGEGG: pH 7.30, GGXGG: pH 7.76, GGXGXGG: pH 7.61, GGXXGG: pH 7.73, O*steocalcin*P15-N26 Gla: pH 7.36, O*steocalcin*P15-N26 Glu: pH 7.26, Conantokin T: pH 7.50. Standard temperature is 14 ^o^C.

| GGEGG | HN | Hα | Hβ | Hγ | Hδ | Hε |
| --- | --- | --- | --- | --- | --- | --- |
| Gly 1 | 8.46 | 3.96 | - | - | - | - |
| Gly 2 | 8.49 | 3.98 | - | - | - | - |
| Glu 3 | 8.63 | 4.30 | 2.08, 1.97 | 2.28 | - | - |
| Gly 4 | 8.69 | 3.97 | - | - | - | - |
| Gly 5 | 8.34 | 3.93 | - | - | - | - |
|  |  |  |  |  |  |  |
| GGXGG |  |  |  |  |  |  |
| Gly 1 | 8.43 | 3.96 | - | - | - | - |
| Gly 2 | 8.50 | 3.98 | - | - | - | - |
| Gla 3 | 8.55 | 4.27 | 2.27, 2.15 | 3.15 | - | - |
| Gly 4 | 8.64 | 3.99 | - | - | - | - |
| Gly 5 | 8.36 | 3.93 | - | - | - | - |
|  |  |  |  |  |  |  |
| GGXXGG |  |  |  |  |  |  |
| Gly 1 | 8.51 | 4.00 | - | - | - | - |
| Gly 2 | 8.56 | 4.01 | - | - | - | - |
| Gla 3 | 8.67 | 4.29 | 2.28, 2.06 | 3.14 | - | - |
| Gla 4 | 8.79 | 4.24 | 2.27, 2.14 | 3.16 | - | - |
| Gly 5 | 8.65 | 3.97 | - | - | - | - |
| Gly 6 | 8.38 | 3.93 | - | - | - | - |
|  |  |  |  |  |  |  |
| GGXGXGG |  |  |  |  |  |  |
| Gly 1 | 8.52 | 3.98 | - | - | - | - |
| Gly 2 | 8.61 | 3.98 | - | - | - | - |
| Gla 3 | 8.72 | 4.25 | 2.27, 2.12 | 3.16 | - | - |
| Gly 4 | 8.52 | 4.03 | - | - | - | - |
| Gla 5 | 8.37 | 4.27 | 2.30, 2.10 | 3.13 | - | - |
| Gly 6 | 8.67 | 3.98 | - | - | - | - |
| Gly 7 | 8.37 | 3.92 | - | - | - | - |
|  |  |  |  |  |  |  |
| O*steocalcin*P15-N26 |  |  |  |  |  |  |
| Pro 15 | - | 4.36 | 2.30, 1.94 | 2.00 | 3.54, 3.50 | - |
| Leu 16 | 8.48 | 4.41 | 1.64, 1.59 | - | 0.93, 0.88 | - |
| Gla 17 | 8.31 | 4.49 | 2.22, 2.04 | 3.18 | - | - |
| Pro 18 | - | 4.40 | 2.27, 1.88 | - | 3.79, 3.74 | - |
| Arg 19 | 8.56 | 4.28 | 1.88, 1.80 | 1.69, 1.63 | 3.24, 3.21 | 7.48 |
| Arg 20 | 8.64 | 4.38 | 1.84, 1.74 | 1.65, 1.60 | 3.20 | 7.33 |
| Gla 21 | 8.71 | 4.29 | 2.25, 2.09 | 3.17 | - | - |
| Val 22 | 8.35 | 4.17 | 2.12 | 0.95, 0.95 | - | - |
| Ser 23 | 8.47 | 4.46 | 3.90, 3.84 | - | - | - |
| Gla 24 | 8.63 | 4.25 | 2.27, 2.10 | 3.18 | - | - |
| Leu 25 | 8.29 | 4.26 | 1.65, 1.58 | - | 0.92, 0.88 | - |
| Asn 26 | 8.47 | 4.68 | 2.85, 2.76 | 7.73, 7.00 (NH2) | - | - |
|  |  |  |  |  |  |  |
| O*steocalcin*P15-N26 |  |  |  |  |  |  |
| Pro 15 | - | 4.36 | 2.29, 1.91 | 1.99 | 3.65, 3.63 | - |
| Leu 16 | 8.44 | 4.35 | 1.64, 1.56 | - | 0.95, 0.87 | - |
| Gla 17 | 8.36 | 4.58 | 2.10, 1.90 | 2.30 | - | - |
| Pro 18 | - | 4.41 | 2.30, 1.88 | 2.50 | 3.80, 3.70 | - |
| Arg 19 | 8.54 | 4.29 | 1.84, 1.78 | 1.68, 1.64 | 3.23, 3.19 | 7.23 |
| Arg 20 | 8.50 | 4.33 | 1.82, 1.76 | 1.65, 1.61 | 3.22, 3.18 | 7.21 |
| Gla 21 | 8.68 | 4.30 | 2.10, 1.97 | 2.24 | - | - |
| Val 22 | 8.35 | 4.13 | 2.10 | 0.87, 0.87 | - | - |
| Ser 23 | 8.47 | 4.44 | 3.88, 3.85 | - | - | - |
| Gla 24 | 8.56 | 4.29 | 2.15, 1.98 | 2.28 | - | - |
| Leu 25 | 8.34 | 4.31 | 1.63, 1.58 | - | 0.94, 0.87 | - |
| Asn 26 | 8.45 | 4.67 | 2.83, 2.76 | 7.68, 6.98 (NH2) | - | - |
|  |  |  |  |  |  |  |
| Conantokin T |  |  |  |  |  |  |
| Gly 1 | - | 4.08, 3.94 | - | - | - | - |
| Glu 2 | 9.20 | 4.21 | 2.12, 2.06 | 2.37 | - | - |
| Gla 3 | 9.30 | 4.15 | 2.22, 2.19 | 3.20 | - | - |
| Gla 4 | 8.23 | 4.05 | 2.41, 2.19 | 3.20 | - | - |
| Tyr 5 | 8.17 | 4.37 | 3.20, 3.12 | - | 7.17 | 6.81 |
| Gln 6 | 8.23 | 3.90 | 2.18, 2.14 | 2.50 | 8.05, 6.86 (NH2) | - |
| Lys 7 | 7.84 | 4.08 | 1.88 | 1.56, 1.51 | 1.70 | 2.97 |
| Met 8 | 7.90 | 4.17 | 2.32, 2.13 | 2.70, 2.48 | - | 2.01 |
| Leu 9 | 7.99 | 3.97 | 1.88, 1.42 | 1.43 | 0.78, 0.74 | - |
| Gla 10 | 8.23 | 4.05 | 2.36, 2.22 | 3.38 | - | - |
| Asn 11 | 8.30 | 4.53 | 2.95, 2.86 | - | 7.69, 6.95 (NH2) | - |
| Leu 12 | 8.14 | 4.19 | 1.84, 1.61 | 1.81 | 0.91, 0.88 | - |
| Arg 13 | 7.98 | 4.11 | 1.98, 1.90 | 1.72, 1.58 | 3.30, 3.20 | 7.77 |
| Gla 14 | 8.33 | 4.09 | 2.27, 2.24 | 3.25 | - | - |
| Ala 15 | 8.13 | 4.17 | 1.50 | - | - | - |
| Glu 16 | 7.94 | 4.13 | 2.13 | 2.34, 2.31 | - | - |
| Val 17 | 7.96 | 3.83 | 2.22 | 1.05, 0.96 | - | - |
| Lys 18 | 8.11 | 4.16 | 1.86 | 1.60, 1.44 | 1.69 | 3.01 |
| Lys 19 | 8.07 | 4.15 | 1.88 | 1.51, 1.42 | 1.69 | 2.97 |
| Asn 20 | 8.26 | 4.66 | 2.88, 2.78 | - | 7.69, 7.06  (NH2) | - |
| Ala 21 | 8.02 | 4.25 | 1.45 | - | 7.47, 7.18  (amidated NH2) | - |

Table S6: ^13^C chemical shifts (ppm) of Gla and Glu containing model peptides at indicated pH values. GGEGG: pH 7.30, GGXGG: pH 7.76, GGXGXGG: pH 7.61, GGXXGG: pH 7.73, O*steocalcin* P15-N26 Gla: pH 7.36, O*steocalcin* P15-N26 Glu: pH 7.26, Conantokin T : pH 7.50 *CO Carbon shifts that were not determined for Conantokin T since these were not visible in the sHMBC spectra.

| GGEGG | CO | Cα | Cβ | Cγ | Cδ | Cε |
| --- | --- | --- | --- | --- | --- | --- |
| Gly 1 | 175.31 | 45.44 | - | - | - | - |
| Gly 2 | 174.73 | 45.17 | - | - | - | - |
| Glu 3 | 177.62 | 57.00 | 29.99 | 36.22 | 184.25 | - |
| Gly 4 | 174.91 | 45.56 | - | - | - | - |
| Gly 5 | 177.08 | 44.92 | - | - | - | - |
|  |  |  |  |  |  |  |
| GGXGG |  |  |  |  |  |  |
| Gly 1 | 175.3 | 45.44 | - | - | - | - |
| Gly 2 | 174.7 | 45.14 | - | - | - | - |
| Gla 3 | 177.6 | 56.37 | 34.13 | 57.78 | 180.84, 180.76 |  |
| Gly 4 | 175.0 | 45.71 | - | - | - | - |
| Gly 5 | 177.1 | 44.95 | - | - | - | - |
|  |  |  |  |  |  |  |
| GGXXGG |  |  |  |  |  |  |
| Gly 1 | - | 45.46 | - | - | - | - |
| Gly 2 | - | 45.22 | - | - | - | - |
| Gla 3 | 177.1 | 55.92 | 34.27 | 57.82 | 181.0, 180.8 | - |
| Gla 4 | 177.6 | 56.48 | 34.17 | 57.72 | 180.9, 180.7 | - |
| Gly 5 | - | 45.76 | - | - | - | - |
| Gly 6 | - | 45.02 | - | - | - | - |
|  |  |  |  |  |  |  |
| GGXGXGG |  |  |  |  |  |  |
| Gly 1 | 175.28 | 45.45 | - | - | - | - |
| Gly 2 | 174.49 | 45.30 | - | - | - | - |
| Gla 3 | 177.41 | 56.46 | 34.29 | 57.85 | 180.9, 180.8 | - |
| Gly 4 | 174.64 | 45.15 | - | - | - | - |
| Gla 5 | 177.55 | 56.03 | 34.37 | 57.74 | 180.8, 180.6 | - |
| Gly 6 | 175.07 | 45.76 | - | - | - | - |
| Gly 7 | 177.11 | 44.96 | - | - | - | - |
|  |  |  |  |  |  |  |
| O*steocalcin* P15-N26 (Gla) |  |  |  |  |  |  |
| Pro 15 | 177.5 | 62.91 | 32.71 | 27.20 | 51.48 | - |
| Leu 16 | 177.5 | 54.64 | 42.48 | 27.03 | 25.03, 23.21 | - |
| Gla 17 | 174.6 | 53.65 | 34.16 | 57.43 | 180.8, 180.5 | - |
| Pro 18 | 176.7 | 62.95 | 32.18 | 27.34 | 50.68 | - |
| Arg 19 | 176.6 | 56.24 | 30.56 | 27.17 | 43.26 | - |
| Arg 20 | 176.2 | 56.06 | 31.09 | 27.36 | 43.29 | - |
| Gla 21 | 176.6 | 56.01 | 34.28 | 57.88 | 181.0, 180.7 | - |
| Val 22 | 176.3 | 62.42 | 32.87 | 21.27, 20.43 | - | - |
| Ser 23 | 174.8 | 58.63 | 63.97 | - | - | - |
| Gla 24 | 176.9 | 56.10 | 34.24 | 57.73 | 180.8, 180.6 | - |
| Leu 25 | 177.6 | 55.93 | 42.21 | 26.92 | 24.82, 23.49 |  |
| Asn 26 | 177.8 | 53.28 | 39.03 | 177.5 | - |  |
|  |  |  |  |  |  |  |
| O*steocalcin* P15-N26 (Glu) |  |  |  |  |  |  |
| Pro 15 | 177.38 | 62.80 | 32.70 | 27.45 | 51.46 | - |
| Leu 16 | 177.38 | 54.83 | 42.14 | 27.15 | 24.85, 23.40 | - |
| Glu 17 | 174.41 | 54.24 | 29.81 | 35.91 | 184.09 | - |
| Pro 18 | 176.81 | 63.08 | 32.16 | 27.18 | 50.72 | - |
| Arg 19 | 176.41 | 56.11 | 30.70 | 27.15 | 43.33 | - |
| Arg 20 | 176.24 | 56.04 | 30.97 | 26.95 | 43.36 | - |
| Glu 21 | 176.61 | 56.79 | 30.21 | 36.17 | 184.16 | - |
| Val 22 | 176.34 | 62.56 | 32.80 | 21.19, 20.52 | - | - |
| Ser 23 | 177.69 | 58.48 | 63.82 | - | - | - |
| Glu 24 | 176.74 | 56.49 | 30.14 | 36.29 | 184.25 | - |
| Leu 25 | 177.34 | 55.65 | 42.31 | 27.17 | 24.85, 23.40 | - |
| Asn 26 | 177.38 | 53.19 | 39.01 | 177.68 | - | - |
|  |  |  |  |  |  |  |
| Conantokin T |  |  |  |  |  |  |
| Gly 1 | N.D.* | 43.61 | - | - |  |  |
| Glu 2 | 178.21 | 59.00 | 29.57 | 36.27 | 183.74 |  |
| Gla 3 | N.D. | 58.60 | 33.11 | 58.49 | 180.96, 180.79 |  |
| Gla 4 | N.D. | 58.76 | 33.72 | 59.37 | 181.26, 180.57 |  |
| Tyr 5 | 177.15 | 61.01 | 38.27 | - | 133.23 | 118.30 |
| Gln 6 | N.D. | 58.30 | 27.93 | 33.30 |  |  |
| Lys 7 | N.D. | 58.50 | 32.01 | 24.93 | 28.78 | 42.13 |
| Met 8 | N.D. | 58.52 | 31.40 | 31.79 |  | 17.00 |
| Leu 9 | N.D. | 57.77 | 41.57 | 26.31 | 25.48, 22.54 |  |
| Gla 10 | N.D. | 58.01 | 33.84 | 57.56 | 181.49, 179.94 |  |
| Asn 11 | N.D. | 55.74 | 38.55 | 176.32 | - |  |
| Leu 12 | 179.16 | 57.30 | 41.97 | 26.74 | 25.39, 23.12 |  |
| Arg 13 | N.D. | 59.00 | 29.88 | 26.86 | - |  |
| Gla 14 | N.D. | 57.60 | 33.76 | 57.67 | 180.80, 179.81 |  |
| Ala 15 | 180.10 | 54.55 | 18.38 | - | - |  |
| Glu 16 | 178.20 | 58.40 | 29.70 | 36.09 | - |  |
| Val 17 | 178.56 | 65.13 | 31.98 | 22.04, 21.20 | - |  |
| Lys 18 | 178.40 | 58.08 | 32.66 | 25.50 | 29.16 | 42.24 |
| Lys 19 | N.D. | 58.07 | 32.69 | 24.80 | 29.31 | 42.13 |
| Asn 20 | 175.30 | 53.84 | 38.84 | 177.04 | - |  |
| Ala 21 | 180.79 | 52.88 | 19.30 | - | - |  |

Table S7: POTENCI predicted Cα chemical shifts of every individual residue in Conantokin T, as well as the list of apo conantokin T chemical shifts and conantokin T with 35.2 mM Ca^2+^chemical shifts (ppm). *POTENCI is unable to predict post-translationally modified amino acids, including γ-carboxyglutamic acid.

| Residue | Conantokin T POTENCI Cα Chemical Shift (ppm) | Conantokin T Apo Cα Chemical Shift (ppm) | Δ chemical shift Apo – POTENCI (ppm) | Conantokin T 35.2 mM Ca^2+^ Cα Chemical Shift (ppm) | Δ chemical shift 35.2 mM Ca^2+^ - Apo (ppm) |
| --- | --- | --- | --- | --- | --- |
| Gly 1 | 45.24 | 43.61 | -1.63 | 43.72 | 0.11 |
| Glu 2 | 56.75 | 59.00 | 2.25 | 59.06 | 0.06 |
| Gla 3 | N.D.* | 58.60 | N.D. | 58.54 | -0.06 |
| Gla 4 | N.D. | 58.76 | N.D. | 58.49 | -0.27 |
| Tyr 5 | 58.08 | 61.01 | 2.93 | 61.15 | 0.14 |
| Gln 6 | 55.70 | 58.30 | 2.60 | 58.76 | 0.46 |
| Lys 7 | 56.55 | 58.50 | 1.95 | 58.52 | 0.02 |
| Met 8 | 55.53 | 58.52 | 2.99 | 58.94 | 0.42 |
| Leu 9 | 55.28 | 57.77 | 2.49 | 57.98 | 0.21 |
| Gla 10 | 56.78 | 58.01 | N.D. | 57.60 | -0.41 |
| Asn 11 | 53.44 | 55.74 | 2.30 | 56.27 | 0.53 |
| Leu 12 | 55.46 | 57.30 | 1.86 | 58.01 | 0.71 |
| Arg 13 | 56.29 | 59.00 | 2.71 | 59.78 | 0.78 |
| Gla 14 | N.D. | 57.60 | N.D. | 57.90 | 0.30 |
| Ala 15 | 52.62 | 54.55 | 1.93 | 54.91 | 0.36 |
| Glu 16 | 56.55 | 58.40 | 1.85 | 58.95 | 0.55 |
| Val 17 | 62.39 | 65.13 | 2.74 | 65.70 | 0.57 |
| Lys 18 | 56.24 | 58.08 | 1.86 | 58.55 | 0.47 |
| Lys 19 | 56.42 | 58.07 | 1.65 | 58.45 | 0.38 |
| Asn 20 | 53.17 | 53.84 | 0.67 | 54.01 | 0.17 |
| Ala 21 | 52.88 | 52.88 | 0.00 | 52.98 | 0.10 |
